# An Initial Diagnosis of Acquired Hemophilia A in an Elderly Male With a Neck Hematoma

**DOI:** 10.7759/cureus.99320

**Published:** 2025-12-15

**Authors:** Elena Quinonez Del Cid, Zachary Elliott, Christopher B Sullivan, Patrick Ellsworth, Doris Lin

**Affiliations:** 1 Otolaryngology - Head and Neck Surgery, University of North Carolina at Chapel Hill School of Medicine, Chapel Hill, USA; 2 Hematology, University of North Carolina at Chapel Hill School of Medicine, Chapel Hill, USA

**Keywords:** acquired hemophilia a, acquired hemophilia a (aha), acquired hemophilia a management, cervical hematoma, head and neck trauma

## Abstract

Acquired hemophilia A (AHA) is a rare hematologic disorder that most commonly presents in the elderly. Hematomas due to AHA are commonly found in the extremities and uncommonly found in the head and neck region. We present an atypical presentation of AHA in an elderly male with a neck hematoma secondary to a ground-level fall. In addition to older age, common associated characteristics with AHA include malignancy and a history of autoimmune disorder. Timely diagnosis of AHA is critical, as delays in treatment can adversely affect outcomes. Initial non-surgical work-up can include laboratory testing with activated partial thromboplastin time and cross-sectional imaging, although these modalities are not diagnostic. Hematologic experts should be consulted as soon as possible if AHA is suspected. Surgical intervention may be necessary to obtain hemostasis depending on the severity of the presentation, and perioperative management should be altered accordingly for hemostatic support if a diagnosis of AHA is present. In cases of cervical hematomas in patients with AHA, otolaryngologic surgeons can aid in management with serial assessments of the airway. Although many coagulopathies can manifest with spontaneous and excessive bleeding, AHA should be suspected in any patient with relevant risk factors, no history of bleeding disorders or anticoagulant use, bleeding in an unusual location, and/or bleeding that is disproportionate to the mechanism of trauma. This case highlights the importance of maintaining diagnostic suspicion for AHA and underscores the need for prompt, coordinated multidisciplinary management of this disease.

## Introduction

Injury to the critical vasculature of the neck, in particular the internal carotid, vertebral, and thyrocervical trunk arteries, is potentially life-threatening due to compromise of cerebral blood supply as well as proximity of these vessels to the airway. Mortality from blunt neck trauma has been reported to be as high as 40% [1]. Blunt neck trauma resulting in cervical vascular injury is rare, with an estimated incidence of 1% of all blunt traumas [2]. Elderly patients and those with comorbidities are likely at a higher risk for death and complications from blunt neck trauma when compared to the general population [2]. One feared complication of neck trauma is hematoma, a collection of blood resulting from broken blood vessels, as it can quickly expand and compromise the airway. Underlying coagulopathies can significantly increase the risk of hematomas and are important to identify in a patient presenting with any mechanism of trauma and spontaneous bleeding. However, diagnosis of coagulopathies can be challenging in the presentation of acute bleeding in a patient with no prior history of a bleeding disorder, as is commonly seen in acquired hemophilia A (AHA) [3]. AHA is a rare and life-threatening bleeding disorder caused by autoantibodies to factor VIII (FVIII) [4]. The exact cause and underlying mechanisms of AHA are not fully understood - an estimated 50% of cases are idiopathic, while the remaining cases have an underlying pathology contributing to autoantibody formation [4]. AHA is associated with older age, immunosuppression, autoimmune disorders, pregnancy, and malignancy [3,4]. Timely diagnosis of AHA is critical to minimize the risk of severe bleeding complications and death [4]. We present a case of a large neck hematoma resulting from a ground-level fall in an elderly male, a rare initial presentation of AHA. We further discuss the clinical reasoning behind the perioperative, hematologic, and surgical management approaches utilized at our institution.

## Case presentation

An 84-year-old male with a past medical history of hypothyroidism, type 2 diabetes, hypertension, basal cell carcinoma, polymyalgia rheumatica, and hyperlipidemia presented to the emergency department with a left neck hematoma after a ground-level fall. His chief complaint was left neck pain. The onset of the hematoma was gradual after the fall, developing over a course of 48 hours prior to the patient presenting to the emergency department. He had no reported personal or family history of a bleeding disorder. He was not on any anticoagulation or medication associated with an increased risk of bleeding. At the time of initial evaluation, the patient’s vital signs were stable, and he was non-toxic appearing. His physical exam was notable for a large hematoma over the left neck with left neck fullness, pain with movement of the left shoulder, and scattered purpura over the bilateral legs. He was breathing comfortably on room air, and his airway was patent.

Comprehensive laboratory exams were obtained, with notable findings (Table 1). Coagulation labs (prothrombin time (PT) and partial thromboplastin time (PTT)) were added in the setting of an expanding hematoma disproportionate to the mechanism of injury. The patient had reported increased bruising in recent months and scattered purpura on his legs, which heightened concern for possible coagulopathy contributing to his presentation. Hematology was consulted on account of elevated activated partial thromboplastin time (aPTT), and additional coagulation tests were ordered. The aPTT mixing study returned at 38.3 seconds, thus indicating normalization, but no incubated test was obtained at that point. Factor IX level showed a mild inhibitory pattern with factor XI levels within normal limits. Factor VIII was low at increasing dilutions, suggesting a true deficiency. Combined with the patient's history above, record of normal PTT in 2018, and factor VIII porcine Bethesda inhibitor assay at 15 (N = <0.5 NBU), the patient was confirmed to have an acquired factor VIII inhibitor deficiency (AHA) and not an inherited factor VIII deficiency.

**Table 1:** Notable values from laboratory examination. PT: prothrombin time; aPTT: activated partial thromboplastin time.

Type	Value	Reference range	Unit
Creatinine	1.38	0.73-1.18	mg/dL
Hemoglobin	10.2	12.9-16.5	g/dL
PT	12.6	9.9-12.6	seconds
Platelet count	220	150-450	10^9^/L
aPTT	70.4	24.8-38.4	seconds
aPTT lupus anticoagulant	116.1	0.0-45.2	seconds
aPTT mixing study	38.3	24.8-38.4	seconds
Factor VIII activity	1.4	56.0-186.0	%
Factor VIII inhibitor assay	15	<0.5	NBU
Dilute viper venom time (DVVT)	42.7	0.0-42.4	seconds
Dilute viper venom confirm	40.8	0.0-42.4	seconds
Dilute Russell viper venom time normalized ratio	1.09	0.00-1.20	

**Figure 1:**
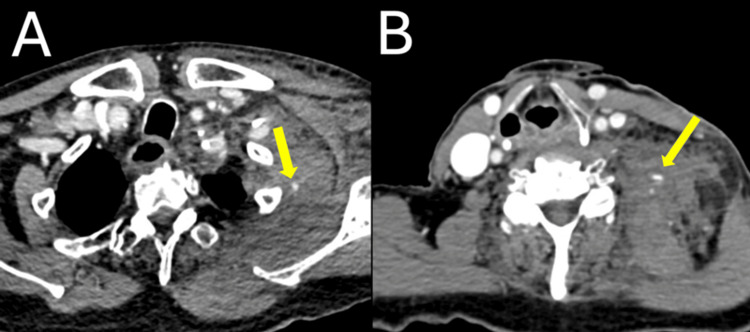
CT of the neck - soft tissue with contrast. This CT demonstrates a large left neck hematoma with extension inferiorly into the left chest wall and axilla with mass effect on adjacent structures. There are foci, indicated by the arrows, concerning for active hemorrhage within the chest wall (panel A) and the left neck (panel B).

**Figure 2:**
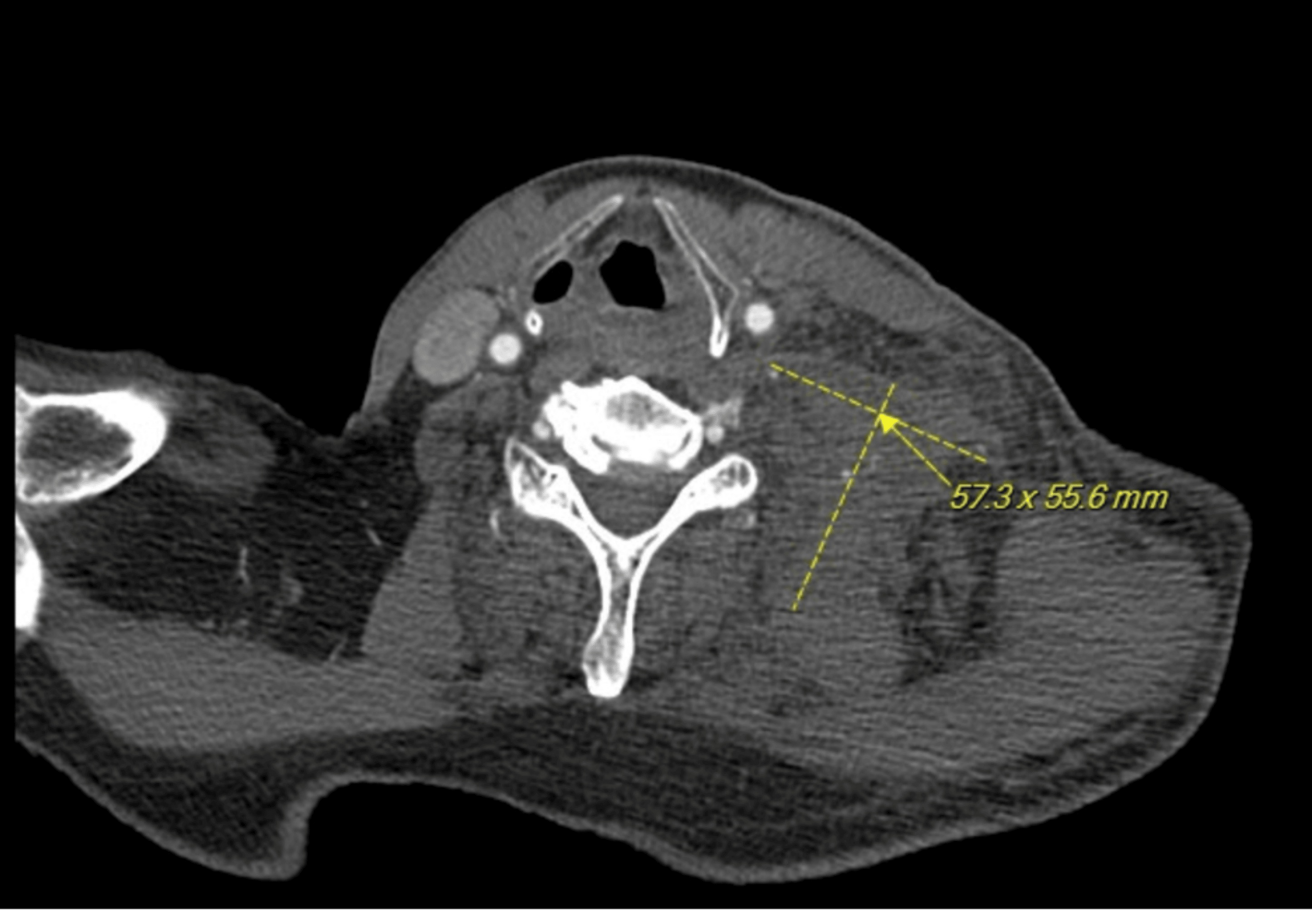
Computed tomography angiography (CTA) of the neck with and without contrast. This CTA demonstrates foci of high-density material within the hematoma (margins indicated by dotted lines) favored to reflect contrast extravasation (indicated by the yellow arrow).

CT of the neck with/without contrast was obtained, which revealed a large hematoma that extended into the chest wall with mass effect concerning for active hemorrhage, indicated by contrast extravasation marked by the yellow arrows (Figure 1). Computed tomography angiography (CTA) of the neck was recommended for further characterization, which revealed similar findings suggestive of active arterial contrast extravasation with unclear vessel of origin, thought to be possibly a branch of the thyrocervical or costocervical arteries (Figure 2). Interventional radiology and otolaryngology - head and neck surgery (OHNS) were contacted for possible intervention.

**Figure 3:**
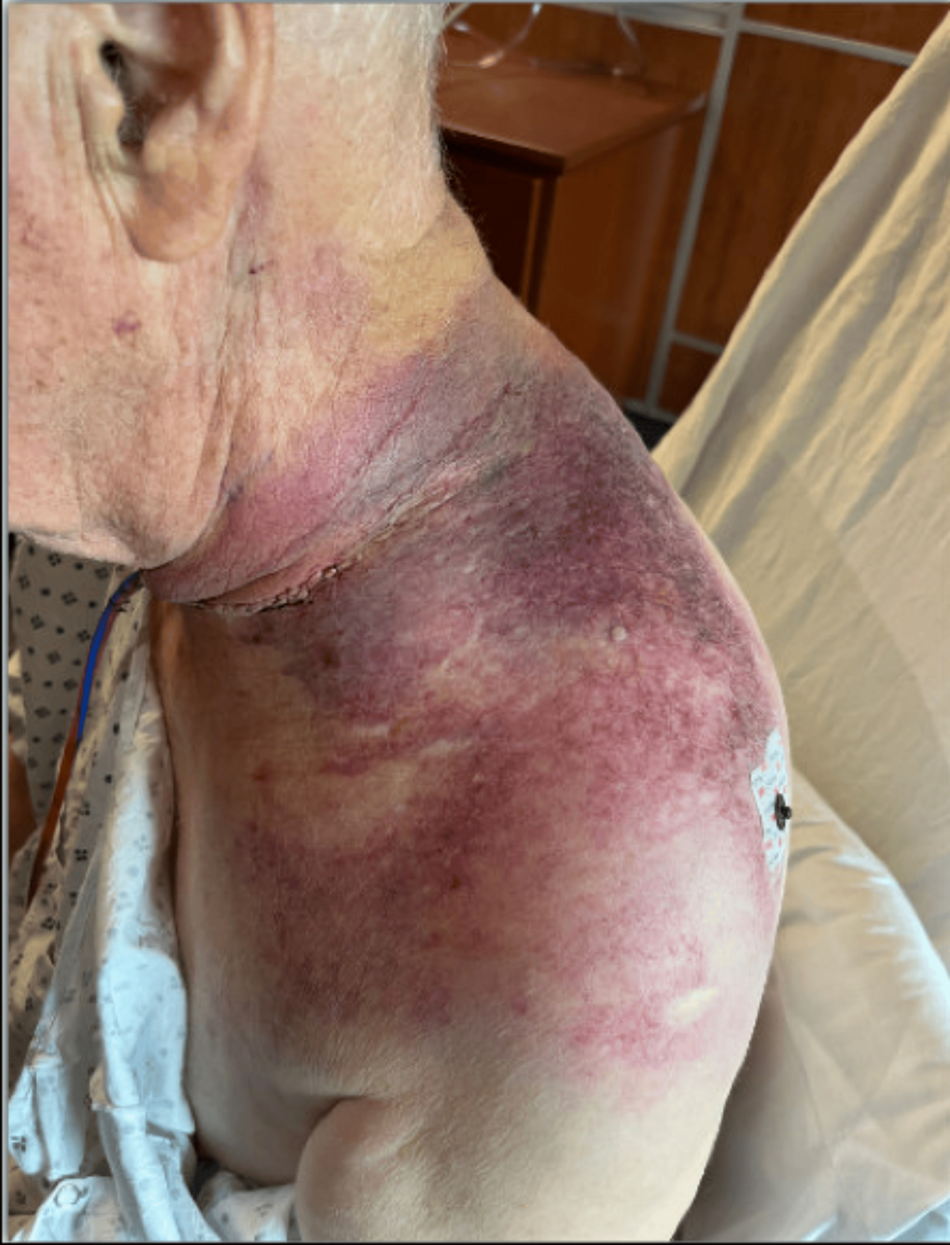
Healing drained hematoma taken on postoperative day one.

The patient underwent a diagnostic cerebral angiogram, which revealed small amounts of contrast extravasation in the distal branches of the costocervical trunk. It was not accessible for embolization. The patient was then taken to the operating room with OHNS for neck exploration and hematoma evacuation. Notable intraoperative findings included bleeding at the level of the thyrocervical trunk and significant tissue edema and ecchymosis of the sternocleidomastoid muscle, omohyoid muscle, and surrounding structures. The hematoma was evacuated from within the sternocleidomastoid muscle and deep within levels III/IV of the neck. The patient tolerated the procedure well and was extubated at the conclusion of the procedure. He was transferred to the floor, and his postoperative course was uneventful (Figure 3).

A hematologic specialist evaluated the patient in the operating room toward the end of the procedure and recommended initiation of IV antihemophilic factor (recombinant) porcine sequence (pFVIII) with an initial dose of approximately 100 units/kg (see Table 2 for exact doses). Factor VIII level was obtained 15 minutes after initial IV pFVIII infusion and was increased to 189%. Factor VIII level the following morning on postoperative day one was 114% and 46.4 units/kg IV pFVIII was given to the patient, with a level at 63% at the time of the second infusion. The patient was then scheduled to receive around 50 units/kg IV pFVIII 12 hours later. IV pFVIII was continued at a dose of roughly 50 units/kg every 12 hours for five days total. IV pFVIII dosing was adjusted accordingly as FVIII trough levels were monitored (Table 2).

**Table 2:** Factor VIII trends throughout the hospitalization in relation to IV recombinant porcine FVIII treatment. POD: postoperative day; FVIII: factor VIII.

Time point	Postoperative day	Time (to the nearest hour)	FVIII level (%)	Notes
Admission	0	6:00 PM	1.40%	Baseline; FVIII severely deficient
15-min post-op porcine FVIII	0	11:00 PM	189%	First recombinant porcine FVIII infusion given at 103 units/kg
POD 1	1	4:00 AM	114%	Overnight monitoring
POD 1	1	10:00 AM	63.00%	Second infusion of recombinant porcine FVIII at 46.4 units/kg
POD 2	2	8:00 AM	80%	Q12 infusions started at a dose of 48.8 units/kg
POD 2	2	8:00 PM	123%	
POD 3	3	10:30 AM	62.00%	
POD 4	4	9:00 AM	76.00%	Q12 infusion dose adjusted to 48.5 units/kg
POD 5	5	9:00 AM	80.00%	Stabilization of FVIII levels at the time of discharge

In terms of initiation of immunosuppressive therapy, rituximab monotherapy was the agent of choice. Hepatitis B serologies were obtained prior to administration of rituximab. After confirming negative hepatitis B serologies, the patient received 1000 mg of rituximab toward the end of his hospital stay, and he was scheduled for outpatient administration of a second dose. He was discharged on prophylactic, off-label treatment for bleeding prophylaxis with emicizumab dosed as 6 mg/kg on day one and 3 mg/kg on day two, followed by 1.5 mg/kg weekly. The patient was scheduled to follow up with hematology weeks following discharge for a more comprehensive hematologic workup and medication management. His factor VIII was corrected to 80% at the time of discharge, and he went home in stable condition.

At the patient’s outpatient hematology visit one month after discharge, AHA diagnosis was confirmed with chromogenic factor VIII assay: inhibitor titer 21 BU (reference range ≤0.5 BU) and chromogenic factor VIII 1.2% (reference range = 55%-200%). He was found to be stable on emicizumab for bleeding prophylaxis and rituximab for immunosuppression with no noted complications.

## Discussion

We present a case of an elderly male with a left neck hematoma that led to a new diagnosis of AHA. AHA is a rare diagnosis, with an estimated incidence of 1.48 per million per year [5]. It is often underrecognized due to limited awareness of the disease, high frequency of concurrent use of anticoagulants in the elderly population, and an often rapidly aggressive and acute presentation, which may hinder timely confirmation [6]. The likelihood of this disorder increases with age, which should heighten suspicion of the diagnosis in a first episode of uncontrolled bleeding in an elderly individual. This patient had a recent history of excessive bruising without the use of anticoagulants, which supported concern of an underlying coagulopathy. In addition to advanced age, this patient had a history of autoimmune disorders (hypothyroidism and polymyalgia rheumatica) frequently associated with AHA.

Although hematomas are a common manifestation of AHA, they are usually found in the upper or lower extremities, rather than the neck [3]. In a single-center retrospective study by Guerrero Camacho et al., 96% of patients with AHA had at least one hematoma, with upper or lower limbs being the most common site (41.3%) and the facial region being amongst the least common (3.5%) [3]. Thus, an initial manifestation of AHA as a neck hematoma is rare. However, the patient had noted increased bruising for months prior to this bleeding episode. This is consistent with the variable presentation of AHA in the literature; this disease can present as anywhere from an incidental finding in an asymptomatic patient to spontaneous, life-threatening bleeding [4]. However, it should be noted that most cases present as major bleeding (defined as a decrease in hemoglobin ≥20 g/L from baseline, transfusion of at least two red blood cell units in 24 hours, bleeding within a critical site, or fatal bleeding) at the time of diagnosis [5]. Very few reported cases of AHA manifesting as hematomas in the head and neck region have been reported [7-10], so there is no clearly defined management plan in the literature. See et al. conducted a comprehensive literature review on head and neck manifestations of AHA from 1950 to 2016 at the time of their case report of a spontaneous cervical bleeding case [9]. They only found nine reported cases of spontaneous hemorrhages in the head and neck region associated with AHA [9]. From 2016 to the present, we have only found four new reported cases of AHA presenting as hematomas in the head and neck region [7-10]. The cases reported have had a wide range of severity, from a small laryngeal hematoma presenting as hoarseness treated with steroids and observation to a spontaneous multilevel airway hemorrhage resulting in airway compromise and urgent tracheostomy [8,9]. The current case is unique in its therapeutic approach: no other reported case amongst those referenced since 2016 has employed pFVIII, rituximab, and emicizumab together for successful management of AHA at the time of diagnosis. This treatment combination has become standard treatment for AHA at our institution, as previously described by Ellsworth et al. [11].

Given the potential severity of bleeding in the cervical region, initial priority upon presentation of a neck hematoma should be the establishment of a secure airway. The role of the otolaryngologic surgeon is to monitor the airway with frequent physical examinations and imaging and obtain hemostasis surgically if indicated. Fortunately, in this case, the patient was hemodynamically stable and did not have airway compromise, but the decision was still made to take him to the operating room due to the expanding nature of the hematoma and risk of acute pharyngeal edema. Prophylactic tracheostomy or intubation was not considered due to the anatomic location of the bleeding, slow expansion of the hematoma, and the patient’s airway patency.

As was done in this case, laboratory analysis, including basic coagulation labs, should be the first step in workup if coagulopathy is suspected. It is critical not to delay diagnosis of AHA, as the mortality rate can be as high as 41% if patients do not receive treatment [12]. Regardless of the presence of laboratory abnormalities, a high suspicion of coagulopathy warrants urgent referral to hematology to guide further workup and management. The diagnosis of AHA frequently requires sophisticated laboratory evaluation best performed by an expert in benign hematology [13]. Screening for AHA usually reveals an isolated prolonged PTT, with a normal PT, platelet count, and thrombin time [13]. It is then important to rule out other causes of a prolonged PTT, such as lupus anticoagulant or use of heparin, which can present as false positives [13]. In this patient, the elevated lupus anticoagulant was deemed a false positive as the patient presented with excessive bleeding (not clotting) along with a normal Dilute Russell viper venom normalized ratio.

The mixing study was corrected in this case, which would not be expected in the presence of inhibitors. However, incubation was not done at the time of diagnosis; therefore, the normalized result could not definitively exclude AHA. The inhibitors in AHA are time- and temperature-dependent and often require incubation at 37°C for at least one hour in order to accurately evaluate inhibitor activity [13]. The time and temperature dependence of the inhibitors is explained by the autoantibodies’ characteristic non-complement-fixing, non-precipitating binding of FVIII [13]. A low FVIII level and elevated Bethesda inhibitor titer, along with the patient’s clinical presentation, confirmed the diagnosis of AHA despite confounding findings of high lupus anticoagulant and a normal mixing study. A chromogenic assay could have been performed during the patient’s hospital stay, but it was not necessary for diagnosis at that time, given the aforementioned findings. Chromogenic inhibitor assays with bovine reagents must be used after initiation of emicizumab, as these assays circumvent confounders and more accurately assess the non-linear kinetics of the FVIII inhibitor [14]. However, an elevated Bethesda inhibitor assay and decreased FVIII levels are sufficient for an initial diagnosis of AHA [14]. A chromogenic assay was later obtained at the patient’s first outpatient visit with hematology one month after discharge, and further confirmed the diagnosis.

Treatment for a hematoma in the setting of AHA varies upon the severity of the presentation but commonly involves hemostatic support and immunosuppression [7-10]. In this case, bleeding was initially controlled with surgery first and recombinant porcine FVIII (pFVIII) second. Ideally, pFVIII or a bypassing agent is administered prior to surgery if deemed appropriate to aid in hemostasis. However, in this case, priority was given to surgical evacuation of the hematoma over initiation of hemostatic therapy prior to surgery due to the threatening proximity of the bleed to the airway.

While pFVIII is not considered a first-line treatment for AHA, evidence has pointed to its efficacy and possibly a lower thromboembolic risk when compared to the typically employed bypassing agents [4]. pFVIII is now the standard treatment for AHA at our institution, with a dosing algorithm that allows successful hemostatic control at lower doses than those used for pFVIII in clinical trials [11]. Despite a higher per-unit cost of pFVIII, the low cumulative doses used to achieve hemostasis, as previously published as a standard at our institution, have enabled overall cost savings [11]. Alternatively, clinicians can opt for bypassing agents (considered first-line) in order to control bleeding; options include recombinant activated factor VII (rFVIIa) or activated prothrombin complex concentrate (aPCC) [4]. Ultimately, the choice of treatment depends on a clinician’s experience, location of bleeding, and resources available [4]. It is important to note that if aPCC is utilized, it should be discontinued and avoided prior to the administration of emicizumab due to the associated increased risk of thrombosis and thrombotic angiopathy when these agents are used together [11].

In terms of immunosuppression, rituximab monotherapy was the hematologic team’s preferred agent for immunosuppression with combined emicizumab therapy for the prevention of future bleeding. This decision was based on clinical experience with patient cases of FVIII inhibitor titers of <20, but still high titers (>5). At our institution, rituximab monotherapy is favored instead of combination therapy with steroids in efforts to avoid the general intolerance to steroids in the elderly and increased risk for infection [11]. For the prevention of recurrence, the use of emicizumab was off-label in this case. Emicizumab is an FVIII mimetic that is increasingly being used off-label for bleeding prophylaxis and perioperative management, but ongoing studies are investigating its utility [15]. Emicizumab was recently approved for AHA in Japan but is still not approved specifically for AHA in the United States or Europe [4]. Pfrepper et al. published recommendations based on clinical consensus that emicizumab is effective for bleed prophylaxis and should be considered from the time of diagnosis [16]. Emicizumab should typically be initiated after the control of acute bleeding and confirmation of no urgent additional procedures or surgeries [11]. Emicizumab for the prevention of bleeding recurrence has been incorporated into the treatment algorithm for AHA at our institution, and ongoing clinical trials are evaluating its efficacy [11].

Because of the rarity and complexity of the diagnosis and management of AHA, confirmation of this condition should be performed in conjunction with hematologic specialists.

## Conclusions

Although it is a rare disorder, otolaryngologists should maintain a high suspicion for AHA in patients presenting with spontaneous bleeding out of proportion to the mechanism of injury in the head and neck region. Hematomas occurring in abnormal locations (i.e., outside of the extremities) or disproportionate to the mechanism of injury should prompt workup of an underlying coagulopathy. Implications of a missed diagnosis of AHA can be fatal, while a timely diagnosis can significantly improve outcomes. Early procurement of coagulation testing and involvement of a hematologic specialist are crucial in the presence of a suspected diagnosis of AHA. In cases of hematomas in the head and neck due to AHA, otolaryngologic surgeons can help in management by continuous assessment of the airway at risk and potential need for surgical intervention to obtain hemostasis. Extensive laboratory testing should be performed to rule out other causes of prolonged PTT, including heparin use, lupus anticoagulant, or disseminated intravascular coagulation. Rapid initiation of medical hemostatic therapy with bypassing agents or recombinant porcine factor VIII is crucial in cases of newly diagnosed AHA. This case highlights the importance of early detection of this diagnosis and multidisciplinary treatment for suspicious, unusual bleeding, particularly in patients with advanced age and a history of autoimmune disease.
